# Assessing uncertainty in the burden of hepatitis C virus: Comparison of estimated disease burden and treatment costs in the UK

**DOI:** 10.1111/jvh.12847

**Published:** 2018-03-01

**Authors:** F. Gubay, R. Staunton, C. Metzig, I. Abubakar, P. J. White

**Affiliations:** ^1^ MRC Centre for Outbreak Analysis and Modelling and NIHR Health Protection Research Unit in Modelling Methodology School of Public Health Imperial College London London UK; ^2^ Department of Mathematics Imperial College London London UK; ^3^ Institute for Global Health University College London London UK; ^4^ Medical Directorate Public Health England London UK; ^5^ MRC Clinical Trials Unit University College London London UK; ^6^ Modelling and Economics Unit National Infection Service Public Health England London UK

**Keywords:** disease burden, hepatitis C virus, Markov model, uncertainty

## Abstract

Hepatitis C virus (HCV) is a major and growing public health concern. We need to know the expected health burden and treatment cost, and understand uncertainty in those estimates, to inform policymaking and future research. Two models that have been important in informing treatment guidelines and assessments of HCV burden were compared by simulating cohorts of individuals with chronic HCV infection initially aged 20, 35 and 50 years. One model predicts that health losses (measured in quality‐adjusted life‐years [QALYs]) and treatment costs decrease with increasing initial age of the patients, whilst the other model predicts that below 40 years, costs increase and QALY losses change little with age, and above 40 years, they decline with increasing age. Average per‐patient costs differ between the models by up to 38%, depending on the patients' initial age. One model predicts double the total number, and triple the peak annual incidence, of liver transplants compared to the other model. One model predicts 55%‐314% more deaths than the other, depending on the patients' initial age. The main sources of difference between the models are estimated progression rates between disease states and rates of health service utilization associated with different disease states and, in particular, the age dependency of these parameters. We conclude that decision‐makers need to be aware that uncertainties in the health burden and economic cost of HCV disease have important consequences for predictions of future need for care and cost‐effectiveness of interventions to avert HCV transmission, and further quantification is required to inform decisions.

**Abbreviations**
DAAs: direct acting antivirals
HCC: hepatocellular carcinoma
NHS: National Health Service
PWID: people who inject drugs
QALYs: quality‐adjusted life‐years
SVR: sustained virologic response

## 1 INTRODUCTION

**Figure 1:**
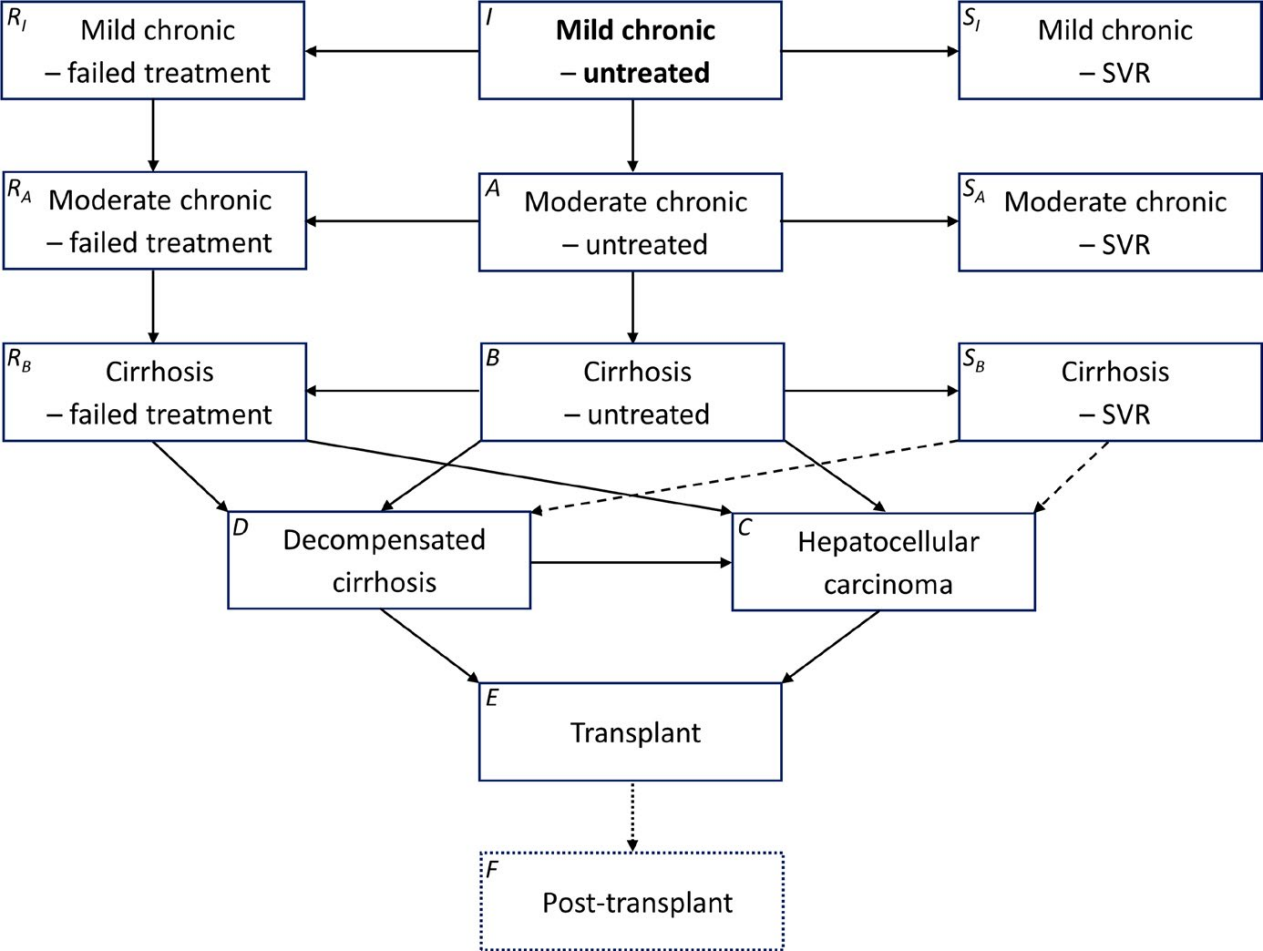
Models describing the progression of chronic hepatitis C virus (HCV) infection. SVR, sustained virologic response. Initially, all individuals are in the state highlighted in bold. Solid lines denote states and progressions occurring in both models. Dashed lines denote progressions occurring in model B only. Dotted lines and compartments denote states and progressions included in model A only. Symbols in the top‐left corners denote the state variables used in the model equations. Both models incorporate state‐ and age‐dependent mortality (not shown for clarity)

The detriment to health and cost to health services caused by liver damage arising from chronic hepatitis C virus (HCV) infection are important determinants of the cost‐effectiveness of interventions to avert HCV transmission, and of treatment strategies targeted at different stages of disease. There is uncertainty in disease progression rates, and health detriment and costs of treatment associated with the different disease stages.^1^ Here, we explore the uncertainty arising from these factors by comparing the models used in two key papers modelling progression of chronic HCV infection. Martin et al.'s model^2^ informed UK guidance,^3^ European recommendations^4^ and an estimate of UK HCV burden,^5^ and Harris et al.'s model^6^ has informed an important review^7^ and an analysis of treatment prioritization.^8^ Here, we compare the two models in terms of estimated health detriment (quality‐adjusted life‐year [QALY] loss) and costs to the health service, using a model structure that can represent both models (Figure 1).

## 2 MATERIALS AND METHODS

We implemented Markov models of HCV progression in a cohort of chronically infected individuals, as described by Martin et al^2^ (“model A”) and Harris et al^6^ (“model B”). We consider a closed cohort and do not consider further transmission of infection or re‐infection of the cohort after treatment/recovery. The models are illustrated in Figure 1 with corresponding sets of equations in Supporting Information. Individuals with mild chronic HCV may progress to moderate disease, cirrhosis, decompensated cirrhosis and hepatocellular carcinoma (HCC), whereas model A uses age‐invariant annual probabilities of progression estimated by Shepherd et al,^9^ and model B has age‐dependent probabilities (Table S2). Individuals with mild disease, moderate disease and cirrhosis receive antiviral treatment of peginterferon‐α and ribavirin at fixed rates. Patients in whom sustained virologic response (SVR) does not occur are not eligible to receive further antiviral treatment.

Parameters specifying rates of progression and treatment are in Table S2,^2^, ^6^, ^10^, ^11^, ^12^ with quality‐of‐life weights and costs in Table S1.^2^, ^13^, ^14^ Cost parameters, which we inflated to 2015‐2016 UK pounds (GBP, £) using the hospital & community health services index,^15^, ^16^ were reported by both Martin et al^2^ and Harris et al,^6^ but only Martin et al^2^ (model A) calculated QALYs and hence reported quality‐of‐life weights. We used these quality‐of‐life weights in model A and model B. Martin et al^2^ (model A) also included health utility values for those in the process of being treated. Whilst the model does not include a compartment for those being treated, it does account for the increased QALY losses and costs for those undergoing treatment. Treatment lasts between 12 and 48 weeks depending on the HCV genotype. A proportion of individuals in mild, moderate and cirrhosis states are treated each year, and therefore, we adjust the QALYs and costs for each of these states accordingly. Rates of age‐dependent annual background mortality were obtained from life tables from the Office for National Statistics,^17^ and quality‐of‐life norms by age were obtained from Kind et al^13^

We considered cohorts of 1000 individuals aged 20, 35 and 50 years at the time of nascent chronic HCV infection, comparing the incidence of progression to various health states and the associated costs and QALY losses predicted by the two models, with results presented per person or per 1000 persons according to ease of reading. The analysis is from the perspective of the National Health Service (NHS) and considered the lifetime of the patient cohort. Health utilities and costs were discounted at 3.5% p.a., as standard for the UK.^18^

The division between HCV genotypes as described in the two models differs, with model A differentiating between genotype 1 and all other genotypes, and model B splitting the genotypes into categories of genotype 1, 4, 5, 6 and genotype 2, 3. To compensate for this, and to better compare between the two models, we split the populations into those who are, or are not, infected with HCV genotype 1 (Table S2).

## 3 RESULTS

### 3.1 Effect of initial age on disease burden

**Figure 2:**
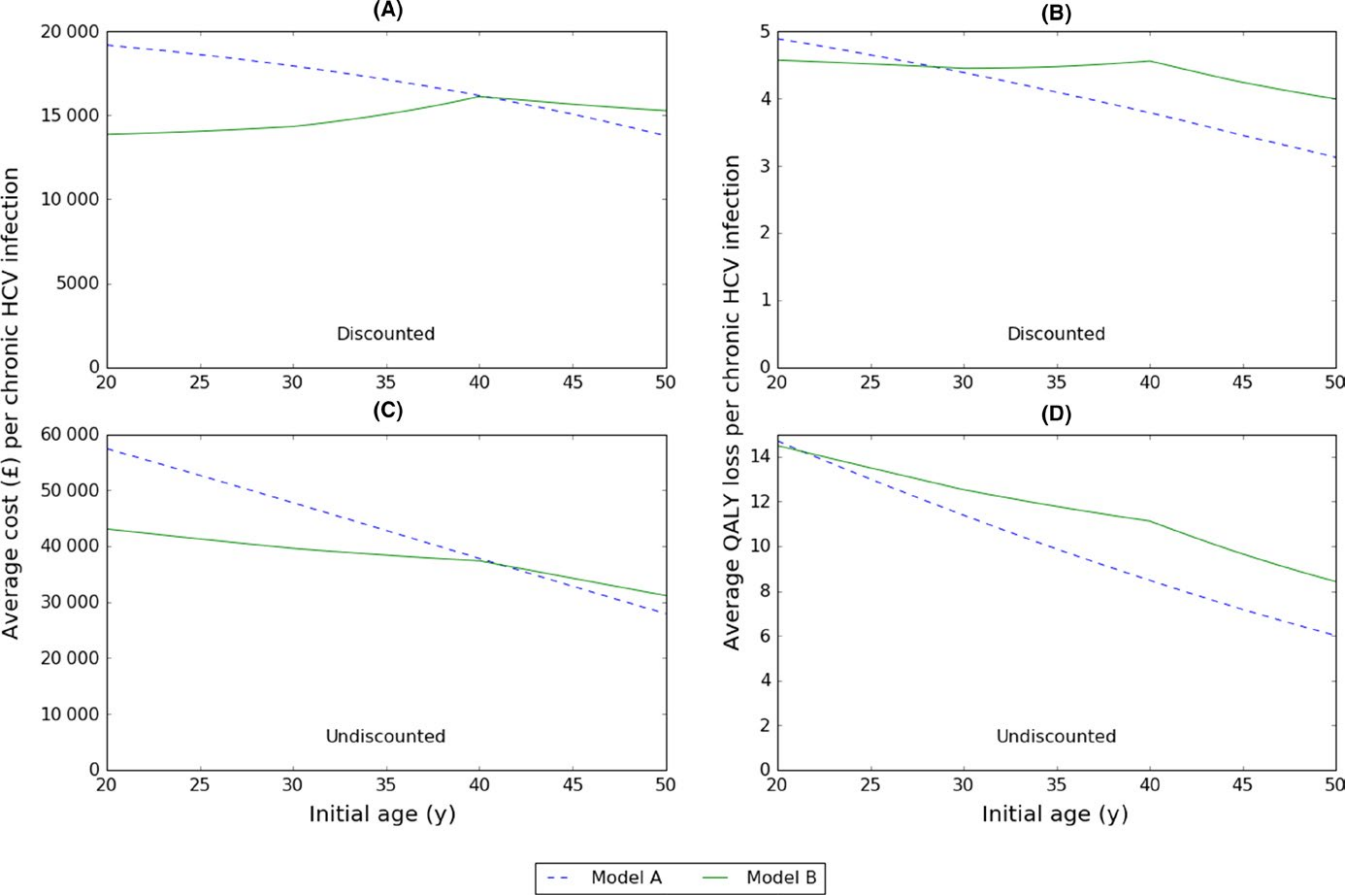
A, Discounted (3.5% p.a.) cumulative costs, (B) discounted (3.5% p.a.) cumulative QALY losses, (C) undiscounted cumulative costs, (D) undiscounted cumulative QALY losses, over the lifetime of the patient by initial age

The two models differ markedly in how initial age affects their predicted burden of HCV (Figure 2). Martin et al.'s model^2^ (“model A” hereafter) predicts monotonically declining discounted costs and QALY losses with increasing initial age (Figure 2A,B), whilst Harris et al's model^6^ (“model B” hereafter) predicts discounted costs increase with initial age below 40 years and then decline slightly (Figure 2A), but that discounted QALY losses change little with initial age below 40 years and then decline (Figure 2B). Model A predicts higher discounted costs than model B except at initial ages >40 years but predicts lower discounted QALY losses except at initial ages <29 years. The age dependency of predicted discounted costs and QALY losses is much greater in model A than model B. As disease progression occurs over decades, discounting has an important effect on the net present value of costs and QALY losses, affecting not only the magnitude but also the effect of initial age: without discounting in both models, the costs and QALYs decline monotonically with increasing initial age, and model A predicts lower QALY losses for all but the very youngest initial ages (Figure 2C,D).

The models differ markedly in the predicted costs, burden of illness (QALY loss) and the incidence of different stages of disease—both in terms of the timing of progression and the numbers of individuals affected, which will affect the need for particular types of care. We examine these differences below.

### 3.2 Incidence of progression to disease states

**Figure 3:**
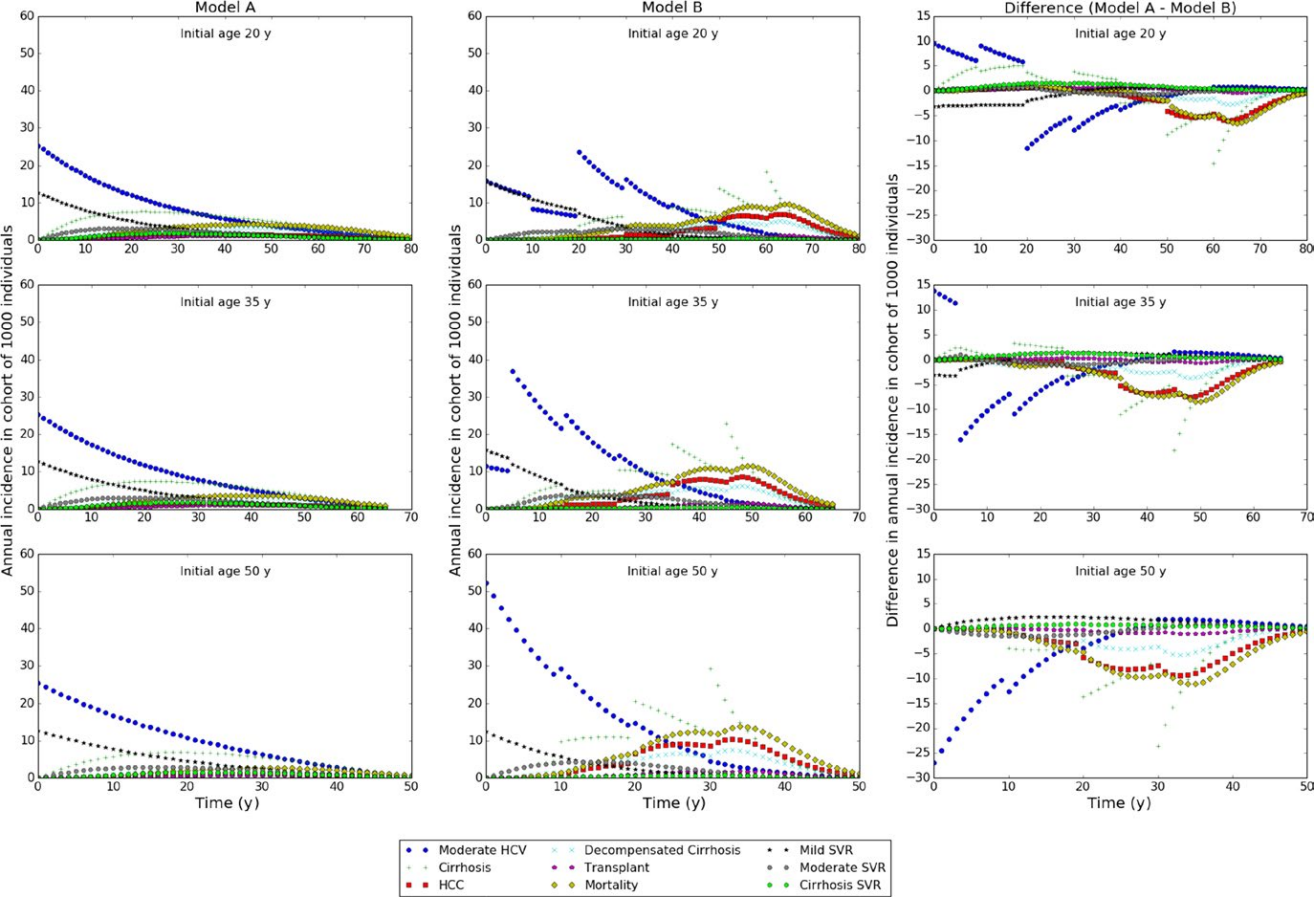
Incidence of disease states in cohorts of 1000 individuals with chronic HCV infection. Top, middle, bottom row: initial age 20, 35 and 50 years, respectively. Columns, left to right: model A, model B, difference (model A—model B)

In model A, the incidence of moderate disease is initially highest and declines monotonically over time, whereas in model B for those initially aged 20 and 35 years, the incidence fluctuates over time and is highest around 40 years of age (ie after 20 years in those initially aged 20 years and after 5 years in those initially aged 35 years), and for those initially aged 50 years, incidence is initially highest and then declines almost monotonically (Figure 3).

There is a striking difference between the models regarding the incidence of cirrhosis and HCC (Figure 3). Trends observed in model A are similar for all initial ages, with incidence of cirrhosis peaking at 7‐8 cases per 1000 infections after roughly 20 years of infection. Incidence of HCC is low (1‐2 cases per 1000 infections per year in all 3 cohorts). In model B, the incidence of cirrhosis and HCC is much higher and has peaks occurring later (around 80 years of age for cirrhosis and a little older for HCC, regardless of initial age), and rates fluctuate over time due to patterns of age dependence.

**Figure 4:**
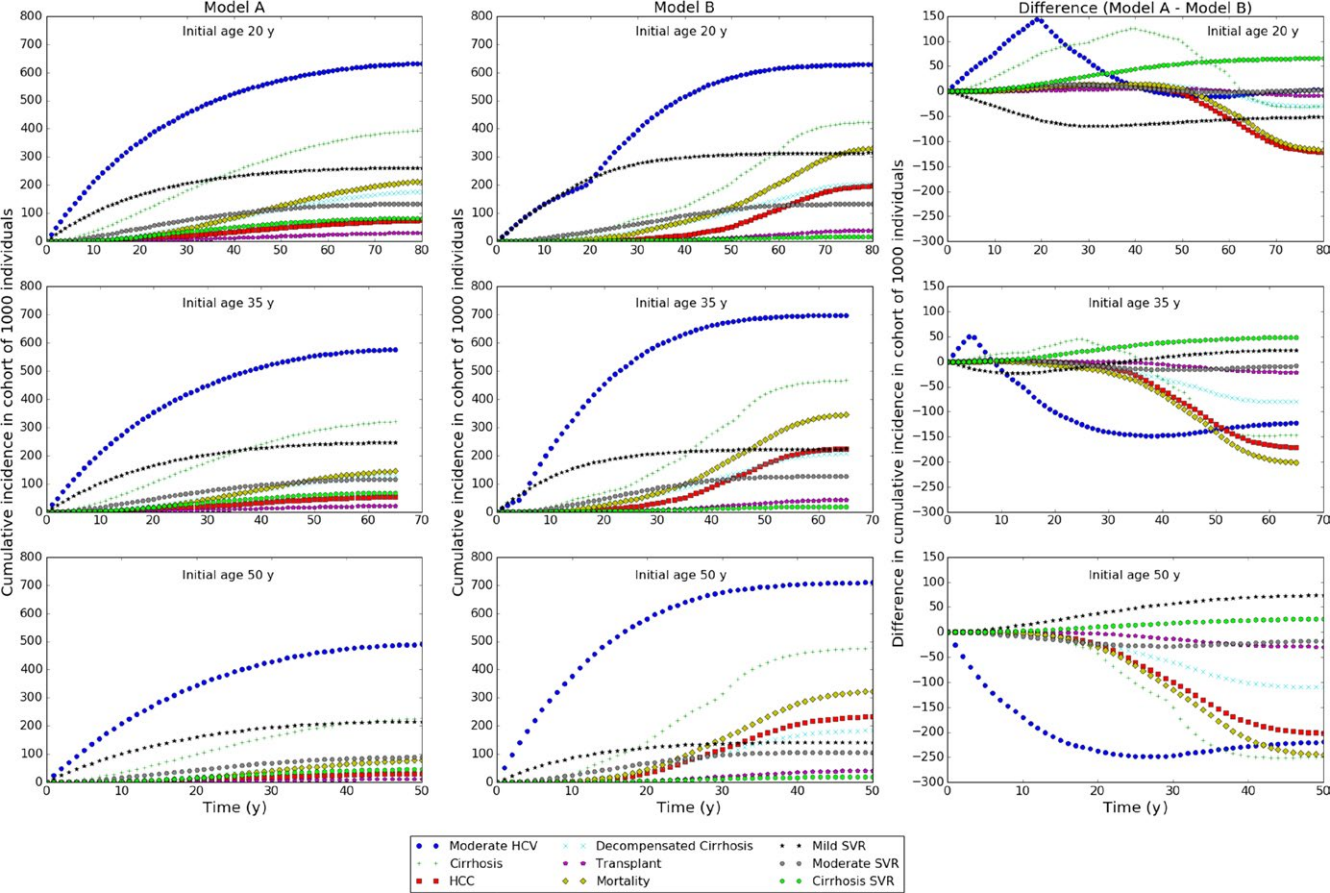
Cumulative incidence of disease states in cohorts of 1000 individuals with chronic HCV infection. Top, middle, bottom row: initial age 20, 35 and 50 years, respectively. Columns, left to right: model A, model B, difference (model A—model B)

Comparison of cumulative incidence makes clear that the models differ not only in the timing of occurrence of disease states but also in the numbers of individuals affected (Figure 4). In those initially aged 20 years, overall numbers of moderate disease cases are similar but they occur earlier in model A. In those initially aged 35 years, more cases of moderate disease occur in the first 5 years in model A, but the number occurring over 10 years is similar and fewer cases occur overall in the lifetime of the patient cohort. In those initially aged 50 years, there are fewer cases in model A at all times. Model A predicts fewer cases of cirrhosis than model B for all initial ages, although for those initially aged 20 years, model A predicts substantial numbers of cirrhosis cases occurring earlier than model B. Model A predicts many fewer cases of HCC than model B, although the two models' predictions of cumulative cases only diverge after age 65 years, regardless of initial age.

Both models predict similar incidence of mild SVR (ie successful treatment among those with mild chronic infection), and in both models, the incidence is initially maximal and then declines monotonically (Figure 3). It should be noted however, that for model A at least, the decrease in SVR over time is a consequence of fewer individuals being in the mild disease state rather than the efficacy of treatment decreasing with age. Cumulative incidence reflects these results, peaking at 261, 245 and 216 cases per 1000 individuals in model A, and 313, 222 and 142 cases in model B. Notice the difference in cumulative incidence is more marked in model B (Figure 4).

The models' SVR predictions are similar, but lower in those with moderate disease, with both models predicting roughly 3 cases per 1000 infections after 15 years of infection in model A, and 10‐30 years in model B for all cohorts (Figure 3). Cumulative incidence decreases with age in both models (A; 134‐89, B; 132‐107) (Figure 4).

Incidence of SVR in those with cirrhosis is higher in model A than in model B, peaking at roughly 2 cases per 1000 infected individuals, compared to less than 1 in model B (Figure 3). Cumulative incidence is much lower in model B than in model A, decreasing from 83 to 46 in model A and increasing from 16 to 19 in model B (Figure 4).

The models differ markedly in the predicted incidence of liver transplants. The total number of transplants occurring in a cohort of 1000 individuals with chronic infection initially aged 20, 35 and 50 years, respectively, is 30, 21 and 11 in model A and 39, 42 and 41 in model B. Notice model A predicts a declining incidence of transplants with increasing age whilst model B predicts no trend. The peak annual frequency of transplants in model B is more than double that of model A, with predictions for the cohort of 1000 individuals with chronic infection initially aged 20, 35 and 50 years, respectively, being 0.6, 0.5 and 0.4 in model A, and 1.2, 1.5 and 1.8 in model B, with the timing of the peak occurring later in model B (Figure 3).

The two models predict different numbers of deaths due to HCV and opposite trends with initial age. Model A predicts numbers of deaths in 1000 individuals with chronic HCV initially aged 20, 35 and 50 years, respectively, being 213, 145 and 78, whilst model B predicts corresponding numbers 330, 345 and 323 (Figure 4). Additionally, the peak incidence of annual mortality is 4.2, 3.7 and 2.7 in model A, and 9.6, 11.5 and 13.7 in model B. The peak occurs earlier in model B (Figure 3), resulting in more life‐years and QALYs lost per death on average.

### 3.3 Health detriment (QALY losses)

The average discounted QALY loss (due to morbidity and mortality) per chronic HCV infection estimated by model A is 4.9, 4.1 and 3.1 for those initially aged 20, 35 and 50 years, respectively (Figure 2B, Table 1). Corresponding discounted QALY losses from model B are 4.6, 4.5 and 4.0, respectively. The models are much more different in their predicted QALY losses due to HCV‐associated mortality. Model A predicts that average discounted QALY losses due to HCV‐related mortality decrease as initial age increases, with 0.81, 0.52 and 0.25 discounted QALYs lost per chronic infection in those initially aged 20, 35 and 50 years, respectively, representing 16.5%, 12.6% and 8.1% of total discounted QALY losses, respectively (Table 1). Model B has a different pattern, with average discounted QALY losses due to HCV‐related mortality being greater in those initially aged 35 years (0.93 per person) than in those initially aged 50 years (0.92), and in those initially aged 20 years (0.76). In model B, the proportion of the total discounted QALY loss that is due to HCV‐related mortality is 16.7%, 20.8% and 23.0% in individuals initially aged 20, 35 and 50 years, respectively (Table 1). Also, with increasing age, there are increases in the magnitude of the differences between model A and B regarding the proportion of QALY losses attributable to different disease stages. Temporal trends in discounted and undiscounted QALY losses in the two models are shown in Figures S1‐S4.

**Table 1:** QALY losses due to morbidity of HCV disease states incurred over the lifetimes of each cohort of 1000 individuals with chronic HCV infection initially aged 20, 35 and 50 years. QALY losses due to mortality are also shown. The percentage of the total QALY loss attributable to morbidity of each disease stage and mortality is also shown. Where applicable, the discount rate used is 3.5% p.a

HCV disease stage	Model A						Model B						QALY–loss differences (model A—model B)		
	QALYs lost			%			QALYs lost			%					
	20 y	35 y	50 y	20 y	35 y	50 y	20 y	35 y	50 y	20 y	35 y	50 y	20 y	35 y	50 y
Discounted															
Mild	1960	1820	1590	40.1	44.4	50.9	1990	1600	1180	43.5	35.7	29.4	−30	220	410
Moderate	948	830	655	19.4	20.3	21.0	966	1150	1200	21.1	25.6	30.0	−18	−320	−545
Cirrhosis	380	311	217	7.8	7.6	6.9	147	211	259	3.2	4.7	6.5	233	100	−42
Decompensated cirrhosis	87	68	45	1.8	1.7	1.4	43	53	54	0.9	1.2	1.4	44	15	−9
HCC	13	10	7	0.3	0.2	0.2	15	27	40	0.3	0.6	1.0	−2	−17	−33
Transplant and post‐transplant	12	9	5	0.3	0.2	0.2	18	26	32	0.4	0.6	0.8	−6	−17	−27
Mild (SVR)	319	261	187	6.5	6.4	6.0	424	289	150	9.3	6.5	3.8	−105	−28	37
Moderate (SVR)	222	168	107	4.5	4.1	3.4	190	174	148	4.2	3.9	3.7	32	−6	−41
Cirrhosis (SVR)	144	103	59	3.0	2.5	1.9	18	21	21	0.4	0.5	0.5	126	82	38
HCV‐related mortality	805	517	253	16.5	12.6	8.1	762	931	918	16.7	20.8	23.0	43	−414	−665
Total	4890	4090	3130				4570	4480	4000				320	−390	−870
Undiscounted															
Mild	3430	3000	2430	23.4	30.4	40.5	3240	2350	1610	22.4	19.9	19.2	190	650	820
Moderate	2450	1910	1300	16.6	19.3	21.6	2840	2670	2130	19.6	22.6	25.2	−390	−760	−830
Cirrhosis	1290	896	511	8.8	9.1	8.5	672	695	636	4.6	5.9	7.6	618	201	−125
Decompensated cirrhosis	333	219	114	2.3	2.2	1.9	180	168	135	1.2	1.4	1.6	153	51	−21
HCC	47	32	17	0.3	0.3	0.3	97	109	110	0.7	0.9	1.3	−50	−77	−93
Transplant and post‐transplant	57	33	14	0.4	0.3	0.2	114	113	96	0.8	1.0	1.1	−57	−80	−82
Mild (SVR)	1070	716	412	7.3	7.3	6.9	1390	751	316	9.6	6.4	3.8	−320	−35	96
Moderate (SVR)	911	552	273	6.2	5.6	4.5	835	593	365	5.8	5.0	4.3	76	−41	−92
Cirrhosis (SVR)	672	377	164	4.6	3.8	2.7	96	83	61	0.7	0.7	0.7	576	294	103
HCV‐related mortality	4440	2140	772	30.2	21.7	12.9	5020	4250	2960	34.6	36.1	35.2	−580	−2110	−2188
Total	14 700	9880	6000				14 500	11 800	8420				200	−1920	−2420

Mild and moderate disease stages account for 59%‐72% of QALY losses in model A and 49%‐65% in model B, with the proportion increasing with initial age in both models (Figure 2B, Table 1). Up to half of all discounted QALY losses in the models are due to mild disease (Table 1). Model A predicts average losses per person of 1.96, 1.82 and 1.59 in the mild stage in those initially aged 20, 35 and 50 years, respectively, representing 40%, 44% and 51% of all discounted QALY losses, respectively. In model B, the corresponding figures are 1.99 (44%), 1.60 (36%) and 1.18 (29%), respectively. In both models, the QALY losses associated with mild disease decline with increasing initial age, whereas the proportion of total QALY loss increases with initial age in model A but declines in model B.

Moderate disease accounts for a large proportion of QALY losses (Table 1). The discounted QALY losses (and proportion of total discounted QALY losses) associated with moderate disease in those initially aged 20, 35 and 50 years are 0.95 (19.4%), 0.83 (20.3%) and 0.66 (21.0%) per person in model A, and 0.97 (21.1%), 1.15 (26.5%) and 1.20 (30.0%) in model B. In both models, the proportion of total discounted QALY loss increases with initial age, whereas the discounted QALY losses decline with increasing initial age in model A but increase in model B.

Quality‐adjusted life‐years losses due to cirrhosis decline with increasing initial age in model A whilst increasing in model B (Table 1). There are also substantial differences in the magnitude of QALY losses. The difference is greatest for those initially aged 20 years, with discounted QALY losses predicted by model A (0.38 per person) being more than double compared to model B (0.15). For individuals initially aged 35 years, model A predicts discounted QALY losses (0.31) that are 47% greater than model B (0.21). In contrast, for individuals initially aged 50 years, model A predicts discounted QALY losses (0.22) that are 16% less than model B (0.26). Similar patterns are seen with decompensated cirrhosis.

Hepatocellular carcinoma causes similar discounted QALY losses in those initially aged 20 years in each model (13 per 1000 individuals in model A, 15 in model B), but in model A, the loss decreases with increasing initial age, whilst in model B it increases, being 10 in model A and 27 in model B in those initially aged 35 years, and 7 in model A and 40 in model B in those initially aged 50 years (Table 1).

### 3.4 Costs

The cumulative discounted cost per chronic HCV infection declines with increasing initial age in model A (£19 200, £17 100 and £13 800 in those initially aged 20, 35 and 50 years, respectively), whereas in model B, costs increase slightly with initial age (£13 900, £15 100 and £15 300 in those initially aged 20, 35 and 50 years, respectively) (Table 2). Temporal trends in discounted and undiscounted costs in the two models are shown in Figures S5‐S8.

**Table 2:** Costs of HCV disease states incurred over the lifetime of the average individual with chronic HCV infection initially aged 20, 35 and 50 years, calculated by each model. The percentage of the total discounted cost attributable to each disease stage is also shown. Where applicable, the discount rate used is 3.5%p.a

	Model A						Model B						Cost differences (model A—model B)		
	Cost (£)			%			Cost (£)			%			(£)		
HCV disease stage	20 y	35 y	50 y	20 y	35 y	50 y	20 y	35 y	50 y	20 y	35 y	50 y	20 y	35 y	50 y
Discounted															
Mild	2520	2450	2270	13.1	14.3	16.4	4280	3580	2780	30.9	23.8	18.1	−1760	−1130	−510
Moderate	3680	3390	2810	19.2	19.8	20.4	4430	5490	5980	32.0	36.4	39.1	−750	−2100	−3170
Cirrhosis	1880	1610	1180	9.8	9.4	8.6	864	1290	1660	6.2	8.5	10.8	1016	320	−480
Decompensated cirrhosis	2650	2190	1490	13.8	12.8	10.8	1330	1680	1790	9.5	11.2	11.7	1320	510	−300
HCC	351	296	209	1.8	1.7	1.5	447	789	1200	3.2	5.2	7.9	−96	−493	−991
Transplant and post‐transplant	442	357	234	2.3	2.1	1.7	428	662	894	3.0	4.4	5.8	14	−305	−660
Mild (SVR)	4050	3800	3370	21.1	22.2	24.4	1680	1190	649	12.1	7.9	4.2	2370	2610	2721
Moderate (SVR)	2210	1910	1460	11.5	11.1	10.6	370	355	311	2.7	2.4	2.0	1840	1555	1149
Cirrhosis (SVR)	1400	1130	763	7.3	6.6	5.5	25	31	32	0.2	0.2	0.2	1375	1099	31
Total	19 200	17 100	13 800				13 900	15 100	15 300				5300	2000	−1500
Undiscounted															
Mild	4550	4150	3530	7.9	9.7	12.6	7000	5280	3810	16.2	13.7	12.2	−2450	−1130	−280
Moderate	9900	8050	5700	17.3	18.8	20.4	13 400	12 900	10 700	31.0	33.7	34.3	−3500	−4850	−5000
Cirrhosis	6690	4800	2840	11.7	11.2	10.2	4040	4300	4090	9.4	11.2	13.1	2650	500	−1250
Decompensated cirrhosis	10 500	7180	3890	18.4	16.8	13.9	5740	5480	4550	13.3	14.3	14.6	4760	1700	−660
HCC	1320	925	525	2.3	2.2	1.9	2840	3220	3290	6.6	8.4	10.5	−1520	−2295	−2765
Transplant and post‐transplant	1870	1220	626	3.3	2.9	2.2	2450	2600	2460	5.7	6.8	7.9	−580	−1380	−1834
Mild (SVR)	9180	7480	5680	16.0	17.5	20.3	5810	3210	1400	13.5	8.4	4.5	3370	4270	4280
Moderate (SVR)	7550	5300	3220	13.2	12.4	11.5	1700	1240	781	4.0	3.2	2.5	5850	4060	2439
Cirrhosis (SVR)	5820	3700	1940	10.1	8.7	6.9	142	126	94	0.3	0.3	0.3	5678	3574	1846
Total	57 400	42 800	27 900				43 100	38 400	31 200				14 300	4400	−3300

Differences between the models are only partially due to the models having different unit costs. When model A's unit costs are used in model B, the predicted discounted costs are higher than using model B's unit costs, being £16 100, £17 300 and £17 300 in those initially aged 20, 35 and 50 years, respectively (Table S3). When using model B's unit costs in model A, the average discounted cost in persons initially aged 20, 35 and 50 years is £16 100, £14 400 and £11 700, respectively (Table S4). Note that the trends with initial age were unchanged in each model.

Mild and moderate disease stages account for only one‐third of the total discounted cost in model A, whilst accounting for majority of the total cost (>57%) in model B (Table 2), particularly in the younger ages. If the unit costs from model A are used in model B (Table S3), then mild and moderate disease stages still account for more than half of the total cost, and if the unit costs from model B are applied to model A (Table S4), then mild and moderate stages account for ~40% of the total cost, which is still much less than in model B.

Costs due to mild disease decrease with increasing initial age in both models, but the proportion of total costs that they represent increases with initial age in model A whilst declining in model B (Table 2). Costs due to moderate disease decrease with increasing initial age in model A and represent a slightly increasing proportion of total costs; in model B, these costs and the proportion of total costs that they represent both increase with increasing initial age (Table 2). These patterns occur regardless of which set of unit costs is used (Table 2, Tables S3 and S4).

The average discounted costs per patient incurred due to cirrhosis, decompensated cirrhosis, HCC and liver transplantation decrease with increasing initial age in model A, as do the proportions of total discounted costs that they represent (Table 2); the opposite is the case in model B. The models differ greatly in the predicted costs due to cirrhosis and decompensated cirrhosis in individuals initially aged 20 years, with the cost in model A being approximately double that in model B. The difference declines with increasing initial age, and in those initially aged 50 years, costs in model A are lower, being 71% and 83% of the costs in model B for cirrhosis and decompensated cirrhosis, respectively. Average costs due to HCC are moderately different in the two models in those initially aged 20 years (£351 in model A, £447 in model B) but markedly different in those initially aged 35 years (£296 in model A, £789 in model B) and very different in those initially aged 50 years (£209 in model A, £1200 in model B). Costs due to transplantation are similar in the two models in those initially aged 20 years (£442 in model A, £428 in model B) but markedly different in those initially aged 35 years (£357 in model A, £662 in model B) and very different in those initially aged 50 years (£234 in model A, £894 in model B).

## 4 DISCUSSION

There are important differences between the models that we compared in the predicted burden of disease and cost to the health service arising from chronic HCV infection. Model A (Martin et al^2^) predicted generally lower average discounted QALY losses per person (3.1‐4.9 vs 4.0‐4.6; Table 1) and higher average discounted costs (£13 800‐£19 200 vs £13 900‐£15 300; Table 2) than model B (Harris et al^6^). The unit cost estimates derived from Harris et al^6^ were slightly lower, except for treatment of mild and moderate disease and cirrhosis (Table S1), but this was not the major reason for the difference in calculated costs. The two models differ markedly in how the initial age affects their results, with progression rates being age‐dependent in Harris et al^6^ but not in Martin et al^2^ (Table S2). Model A predicts that costs and QALY losses decline with increasing initial age whilst model B predicts little change or increases with increasing initial age up to 40 years, followed by declines with increasing age. In both models, a large proportion of the costs of HCV infection are due to the mild and moderate disease stages, which involve a large proportion of the infected individuals and have long average duration. Model B predicts much greater numbers of liver transplants, which is important given the limited availability of livers for transplant, and the increasing need for transplants due to obesity‐ and alcohol‐related liver damage.

The uncertainties highlighted by our analysis have important consequences for UK public health planning regarding HCV, as they affect the magnitude and timing of the expected demand for care over the coming years and decades, and the cost‐effectiveness of interventions to against HCV infection and disease. Current NICE guidelines^3^ are based on Martin et al's model,^2^ which is based on the work of Shepherd et al^9^ Importantly, we have shown that Harris et al's model,^6^ with estimates of progression rates that are age‐dependent, produces different estimates of costs and QALY losses due to chronic HCV. As Harris et al's analysis^6^ found that rates differ by age, this should be considered in future modelling analyses.

A strength of this study is its systematic comparison of the models, examining the importance of age‐dependent progression rates and different estimates of unit costs in contributing to uncertainty. The marked differences between the models of Martin et al^2^ and Harris et al^6^ are due primarily to differences in progression rates, which determine numbers of individuals in each disease state, and differences in rates of health service utilization associated with each disease state, rather than differences in estimated unit costs to the health service of treating each health state. Further study of progression rates—and factors associated with variation, such as age—and rates of health service utilization associated with different stages of HCV‐associated disease is required.

There are other important gaps in knowledge that require further empirical study. As progression from chronic HCV infection through disease states can take decades, the life expectancy of infected individuals could be an important determinant of the cost‐effectiveness of treatment. People who inject drugs (PWID) are an important risk group for HCV infection and make up the majority of those infected with HCV in the UK.^15^, ^16^ Treatment as Prevention (TasP) has been advocated for this group, although there are important uncertainties in its likely effectiveness, which depends upon the structure of the network of injecting partners, the coverage of testing and proportion of infected patients who are treated and cured, and the frequency of behaviour change preventing re‐infection after successful treatment.^21^ Further studies of all of these are required, including analysis of potential synergistic effects^22^ of combining HCV TasP with needle and syringe programmes (which reduce the infection risk of injecting) and opiate substitution therapy (which assist PWID in stopping injecting). Use of transmission dynamic models to analyse surveillance data is important in assessing population‐level impacts of interventions;^23^ in the case of PWID, it is important to use models that account for the injecting network structure and dynamics.^21^ In some marginalised populations, proactive “find and treat” services may be cost‐effective or even cost‐saving, as has been found for tuberculosis.^24^, ^25^ Past history of drug injection may reduce life expectancy independent of HCV infection; if so, then this would reduce the cost‐effectiveness of treatment for chronic HCV in that patient group. However, if comorbidities associated with a history of drug injection increase rates of progression to later‐stage disease, then this would tend to increase cost‐effectiveness of treatment. Factors that should also be taken into consideration when calculating the cost‐effectiveness of treatment are alcohol consumption, gender and co‐infection, as each have been found to vary HCV disease progression rates,^26^, ^27^ and also affect life expectancy. We recommend studies of patient health records to determine the rates of health service utilization—both primary and secondary care—by patients in different stages of disease and with different comorbidities. Importantly, actual patient pathways can be different from idealised pathways typically used in economic evaluations, and there can be considerable variation between patients (eg see Ref. 28). Finally, the impact on disease progression and health service utilization in individuals treated with direct acting antivirals (DAAs), which are highly efficacious and well‐tolerated but are also expensive, requires further study to determine how to use DAAs in a cost‐effective manner. However, if the price paid for DAAs is too high, then this damages overall population health because the money could be used in another way to obtain a greater health gain.

In summary, this research has emphasised the uncertainty in the health burden and economic cost of HCV disease in the UK. The main source of uncertainty is progression rates between disease states and associated rates of health service utilization, particularly with regard to age dependency. In addition, the effects of other risk factors and comorbidities require quantification to improve information for planning and decision‐making.

## CONFLICT OF INTEREST

The authors have no conflict of interests to disclose.

## AUTHORS' CONTRIBUTIONS

The models were coded by FG and analysed by FG, RS and PW, after preliminary analysis by CM. The initial draft of the manuscript was written by RS, FG and PW and revised by all authors.
