# Supplementary material for: Assessing uncertainty in the burden of hepatitis C virus: Comparison of estimated disease burden and treatment costs in the UK
Source: J Viral Hepat. 2018 Mar 1;25(5):514–23. doi: 10.1111/jvh.12847 (PMC5947569; doi:10.1111/jvh.12847)

**SUPPORTING INFORMATION**

This document contains tables and figures of results of economic analysis without discounting. The contents are as follows.

**Equations.**

**Table S1**. Health economic parameters of the models.

**Table S2**. Parameter values describing disease progression rates from Martin et al. [2] (model A) and Harris et al. [6] (model B) and SVR probabilities from Thomson et al. [10]. Several parameters in model B are stratified by age-groups, with age-ranges specified in years.

**Table S3**. Costs of HCV disease states incurred over the lifetime of the average individual with chronic HCV infection initially aged 20, 35, and 50 years, calculated by each model with model A unit costs used in both models. Where applicable the discount rate used is 3.5%p.a.

**Table S4**. Costs of HCV disease states incurred over the lifetime of the average individual with chronic HCV infection initially aged 20, 35, and 50 years, calculated by each model with model B unit costs used in both models. Where applicable the discount rate used is 3.5%p.a.

**Figure** **S1**. Discounted QALY losses in cohorts of 1,000 individuals with chronic HCV infection. Top, middle, bottom row: initial age 20, 35, 50 years, respectively. Columns, left to right: model A, model B, difference (model A – model B). Discount rate: 3.5%p.a.

**Figure** **S2**. Undiscounted QALY losses in cohorts of 1,000 individuals with chronic HCV infection. Top, middle, bottom row: initial age 20, 35, 50 years, respectively. Columns, left to right: model A, model B, difference (model A – model B).

**Figure** **S3**. Cumulative discounted QALY losses in individuals with chronic HCV infection. Top, middle, bottom row: initial age 20, 35, 50 years, respectively. Columns, left to right: model A, model B, difference (model A – model B). Discount rate: 3.5%p.a.

**Figure** **S4**. Cumulative undiscounted QALY losses in individuals with chronic HCV infection. Top, middle, bottom row: initial age 20, 35, 50 years, respectively. Columns, left to right: model A, model B, difference (model A – model B).

**Figure** **S5**. Average discounted costs per individual with chronic HCV infection. Top, middle, bottom row: initial age 20, 35, 50 years, respectively. Columns, left to right: model A, model B, difference (model A – model B). Discount rate: 3.5%p.a.

**Figure** **S6**. Average undiscounted costs per individual with chronic HCV infection. Top, middle, bottom row: initial age 20, 35, 50 years, respectively. Columns, left to right: model A, model B, difference (model A – model B).

**Figure** **S7**. Average cumulative discounted costs per individual with chronic HCV infection. Top, middle, bottom row: initial age 20, 35, 50 years, respectively. Columns, left to right: model A, model B, difference (model A – model B). Discount rate: 3.5%p.a.

**Figure** **S8**. Average cumulative undiscounted costs per individual with chronic HCV infection. Top, middle, bottom row: initial age 20, 35, 50 years, respectively. Columns, left to right: model A, model B, difference (model A – model B).

**Equations 1**, describing model A:

Where numbers of individuals in each health state are as follows: $I$: mild HCV infection; $A$: moderate HCV infection; $B$: cirrhosis; $C$: hepatocellular carcinoma; $D$: decompensated cirrhosis; $E$: pre or current liver transplant phase; $F$: post liver transplant; $G$: deceased; $S_{I}$: mild HCV infection with SVR; $S_{A}$: moderate HCV infection with SVR; $S_{B}$: cirrhosis with SVR; $R_{I}$: mild HCV infection that did not respond to treatment; $R_{A}$: moderate HCV infection that did not respond to treatment; $R_{B}$: cirrhosis that did not respond to treatment. The transition-rate parameters are denoted $m$ with some subscript and are derived from transition probabilities denoted $p$ with the corresponding subscript; $m_{A}$: mild HCV infection to moderate HCV infection; $m_{B}$: moderate HCV infection to cirrhosis; $m_{C}$: cirrhosis to hepatocellular carcinoma; $m_{DC}$: decompensated cirrhosis to hepatocellular carcinoma; $m_{DE}$: decompensated cirrhosis to a pre- or current liver transplant phase; $m_{CE}$: hepatocellular carcinoma to a pre- or current liver transplant phase; $m_{F}$: a pre or current liver transplant phase to a post liver transplant phase; $m_{CG}$: hepatocellular carcinoma to death; $m_{DG}$: decompensated cirrhosis to death; $m_{EG}$: a pre or current liver transplant phase to death; $m_{FG}$: a post liver transplant phase to death. Treatment rates are also transition parameters: $m_{\delta_{I}}$: mild HCV; $m_{\delta_{A}}$: moderate HCV; $m_{\delta_{B}}$: cirrhosis; $m_{S_{I}}$, $m_{R_{I}}$: rates of SVR and treatment non-response respectively for mild HCV, where $m_{S_{I}}=m_{\delta_{I}}q_{S_{I}}$, $m_{R_{I}}=m_{\delta_{I}}q_{R_{I}}$; $m_{S_{A}}$, $m_{R_{A}}$: rates of SVR and treatment non-response respectively for moderate HCV, where $m_{S_{A}}=m_{\delta_{A}}q_{S_{A}}$, $m_{R_{A}}=m_{\delta_{A}}q_{R_{A}}$; $m_{S_{B}}$, $m_{R_{B}}$: rates of SVR and treatment non-response for cirrhosis, where $m_{S_{B}}=m_{\delta_{B}}q_{S_{B}}$, $m_{R_{B}}=m_{\delta_{B}}q_{R_{B}}$. The probabilities of successful treatment $q_{S}$and failed treatment $q_{R}$for each individual in a state that undergoes treatment are defined in Equations 3. The background mortality rate is $n$.

**Equations 2**, describing model B:

Where numbers of individuals in each health state are as follows;$I$: mild HCV infection;$A$: moderate HCV infection;$B$: cirrhosis;$C$: hepatocellular carcinoma;$D$: decompensated cirrhosis;$E$: pre or current liver transplant phase;$G$: deceased;$S_{I}$: mild HCV infection with SVR;$S_{A}$: moderate HCV infection with SVR;$S_{B}$: cirrhosis with SVR; $R_{I}$: mild HCV infection that did not respond to treatment;$R_{A}$: moderate HCV infection that did not respond to treatment;$R_{B}$: cirrhosis that did not respond to treatment. The transition probability parameters are denoted$p$with some subscript;$p_{A}$: mild HCV infection to moderate HCV infection;$p_{B}$: moderate HCV infection to cirrhosis;$p_{C}$: cirrhosis to hepatocellular carcinoma;$p_{DC}$: decompensated cirrhosis to hepatocellular carcinoma;$p_{DE}$: decompensated cirrhosis to a pre or current liver transplant phase;$p_{CE}$: hepatocellular carcinoma to a pre or current liver transplant phase;$p_{\delta_{BC}}$: cirrhosis with SVR to hepatocellular carcinoma;$p_{\delta_{BD}}$: cirrhosis with SVR to decompensated cirrhosis;$p_{CG}$: hepatocellular carcinoma to death;$p_{DG}$: decompensated cirrhosis to death;$p_{FG}$: from a post liver transplant phase to death. Treatment rates are also transition parameters;$p_{\delta_{I}}$: mild HCV;$p_{\delta_{A}}$: moderate HCV;$p_{\delta_{B}}$: cirrhosis;$p_{S_{I}},p_{R_{I}}$: rates of SVR and treatment non-response respectively for mild HCV, where $p_{S_{I}}=p_{\delta_{I}}q_{S_{I}}$, $p_{R_{I}}=p_{\delta_{I}}q_{R_{I}}$; $p_{S_{A}},p_{R_{A}}$: rates of SVR and treatment non-response respectively for moderate HCV, where $p_{S_{A}}=p_{\delta_{A}}q_{S_{A}}$, $p_{R_{A}}=p_{\delta_{A}}q_{R_{A}}$;$p_{S_{B}},p_{R_{B}}$: rates of SVR and treatment non-response respectively for cirrhosis, where $p_{S_{B}}=p_{\delta_{B}}q_{S_{B}}$, $p_{R_{B}}=p_{\delta_{B}}q_{R_{B}}$. The probabilities of successful treatment $q_{S}$and failed treatment $q_{R}$for each individual in a state that undergoes treatment are defined in Equations 3. The background mortality rate is$p_{n}$.

**Equations 3**, the probabilities of responding to treatment $q_{S_{I}},q_{S_{A}},q_{S_{B}}$, and not responding to treatment $q_{R_{I}},q_{R_{A}},q_{R_{B}}$for model A are:

$$\begin{matrix} q_{S_{I}}=p_{geno1}p_{SVR(geno1)}+(1-p_{geno1})p_{SVR(genonot1)}, \\ q_{S_{A}}=p_{geno1}p_{SVR(geno1)}+(1-p_{geno1})p_{SVR(genonot1)}, \\ q_{S_{B}}=p_{geno1}p_{SVR(geno1)}+(1-p_{geno1})p_{SVR(genonot1)}, \\ q_{R_{I}}=p_{geno1}(1-p_{SVR(geno1)})+(1-p_{geno1})(1-p_{SVR(genonot1)})=1-q_{S_{I}}, \\ q_{R_{A}}=p_{geno1}(1-p_{SVR(geno1)})+(1-p_{geno1})(1-p_{SVR(genonot1)})=1-q_{S_{A}}, \\ q_{R_{B}}=p_{geno1}(1-p_{SVR(geno1)})+(1-p_{geno1})(1-p_{SVR(genonot1)})=1-q_{S_{B}}. \end{matrix}$$

The probabilities of responding to treatment $q_{S_{I}},q_{S_{A}},q_{S_{B}}$, and not responding to treatment $q_{R_{I}},q_{R_{A}},q_{R_{B}}$for model B are:

$$\begin{matrix} q_{S_{I}}(t)=p_{geno1}p_{SVR(geno1)(I)}(t)+[1-p_{geno1}]p_{SVR(genonot1)(I)}(t), \\ q_{S_{A}}(t)=p_{geno1}p_{SVR(geno1)(A)}(t)+[1-p_{geno1}]p_{SVR(genonot1)(A)}(t), \\ q_{S_{B}}(t)=p_{geno1}p_{SVR(geno1)(B)}(t)+[1-p_{geno1}]p_{SVR(genonot1)(B)}(t), \\ q_{R_{I}}(t)=p_{geno1}[1-p_{SVR(geno1)(I)}(t)]+[1-p_{geno1}][1-p_{SVR(genonot1)(I)}(t)]=1-q_{S_{I}}(t), \\ q_{R_{A}}(t)=p_{geno1}[1-p_{SVR(geno1)(A)}(t)]+[1-p_{geno1}][1-p_{SVR(genonot1)(A)}(t)]=1-q_{S_{A}}(t), \\ q_{R_{B}}(t)=p_{geno1}[1-p_{SVR(geno1)(B)}(t)]+[1-p_{geno1}][1-p_{SVR(genonot1)(B)}(t)]=1-q_{S_{B}}(t). \end{matrix}$$

**Table S1**. Health economic parameters of the models.

| **Quality-of-life weights** | **Value** | | **Reference** |
| --- | --- | --- | --- |
| Uninfected | Age-dependent | | [13] |
| Mild disease | 0.77 | | [2] |
| Moderate disease | 0.66 | |  |
| Cirrhosis | 0.55 | |  |
| Decompensated cirrhosis | 0.45 | |  |
| HCC | 0.45 | |  |
| Liver transplant | 0.45 | |  |
| Post-transplant | 0.67 | |  |
| Treatment – mild | 0.66 | |  |
| Treatment – moderate | 0.55 | |  |
| Treatment – cirrhosis | 0.44 | | [14] |
| SVR – mild | 0.82 | | [2] |
| SVR – moderate | 0.72 | |  |
| SVR – cirrhosis | 0.60 | | [14] |
| **Costs** | **Model A** | **Model B** |  |
| Mild¹ | 182 | 180 | [2,6] |
| Moderate¹ | 947 | 945 |  |
| Cirrhosis¹ | 1,503 | 1,484 |  |
| Decompensated cirrhosis¹ | 12,049 | 11,943 |  |
| HCC¹ | 10,737 | 10,519 |  |
| Liver transplant and first year post transplant care² | 48,603 | 48,159 |  |
| Post-transplant care past 1 year¹ | 1,830 | 1,790 |  |
| Treatment of mild disease² | 11,082 | 12,667 |  |
| Treatment of moderate disease² |  | 12,508 |  |
| Treatment of cirrhosis² |  | 12,463 |  |
| Mild SVR¹ | 342 | 337 |  |
| Moderate SVR¹ | 947 |  |  |
| Cirrhosis SVR¹ | 1,503 |  |  |
| ¹ Costs per year, ²One-off costs | | | |

**Table S2**. Parameter values describing disease progression probabilities from Martin et al. [2] (model A) and Harris et al. [6] (model B) and SVR probabilities from Thomson et al. [10]. Several parameters in model B are stratified by age-groups, with age-ranges specified in years.

| **Parameter** | **Symbol** | **Model A** | **Model B** |
| --- | --- | --- | --- |
| Transition probability per year – mild disease to moderate disease | $p_{A}$ | 0.025 | 20-29: 0.0158 |
|  |  |  | 30-39: 0.0115 |
|  |  |  | 40-49: 0.0425 |
|  |  |  | 50-59: 0.0523 |
|  |  |  | 60-69: 0.0593 |
|  |  |  | 70-79: 0.0667 |
|  |  |  | 80+: 0.0546 |
| Transition probability per year – moderate disease to cirrhosis | $p_{B}$ | 0.037 | 20-29: 0.0074 |
|  |  |  | 30-39: 0.0168 |
|  |  |  | 40-49: 0.0261 |
|  |  |  | 50-59: 0.0142 |
|  |  |  | 60-69: 0.0293 |
|  |  |  | 70-79: 0.0563 |
|  |  |  | 80+: 0.1355 |
| Transition probability per year – cirrhosis to hepatocellular carcinoma | $p_{C}$ | 0.014 | 20-29: 0.0068 |
|  |  |  | 30-39: 0.0113 |
|  |  |  | 40-49: 0.0186 |
|  |  |  | 50-59: 0.0974 |
|  |  |  | 60-69: 0.0851 |
|  |  |  | 70+: 0.0748 |
| Transition probability per year – cirrhosis to decompensated cirrhosis | $p_{D}$ | 0.039 | 20-29: 0.1490 |
|  |  |  | 30-39: 0.1286 |
|  |  |  | 40-49: 0.1123 |
|  |  |  | 50-59: 0.0974 |
|  |  |  | 60-69: 0.0851 |
|  |  |  | 70+: 0.0748 |
| Transition probability per year – decompensated cirrhosis to hepatocellular carcinoma | $p_{DC}$ | 0.014 | 20-29: 0.0114 |
|  |  |  | 30-39: 0.0186 |
|  |  |  | 40-49: 0.0305 |
|  |  |  | 50-59: 0.0502 |
|  |  |  | 60-69: 0.0835 |
|  |  |  | 70+: 0.1371 |
| Transition probability per year – cirrhosis SVR to decompensated cirrhosis | $p_{\delta_{BD}}$ | N/A | 0.0016 |
| Transition probability per year – cirrhosis SVR to hepatocellular carcinoma | $p_{\delta_{BC}}$ | N/A | 0.0021 |
| Transition probability per year – hepatocellular carcinoma to transplant | $p_{CE}$ | 0.03 | 0.1 |
| Transition probability per year – decompensated cirrhosis to transplant | $p_{DE}$ | 0.03 | 0.025 |
| Transition probability per year – decompensated cirrhosis to death | $p_{DG}$ | 0.13 | 0.293 |
| Transition probability per year – hepatocellular carcinoma to death | $p_{CG}$ | 0.43 | 0.6026 |
| Transition probability per year – transplant to death | $p_{EG}$ | 0.21 | 0.083 [11] |
| Transition probability per year – post-transplant to death | $p_{FG}$ | 0.057 | N/A |
| Probability of SVR when treated – genotype 1 | $p_{SVR(1)}$ | 0.45 | N/A |
| Probability of SVR when mild disease treated – genotype 1 | $p_{SVR(I,1)}$ | N/A | 20-39: 0.72 |
|  |  |  | 40-49: 0.57 |
|  |  |  | 50+: 0.40 |
| Probability of SVR when moderate disease treated – genotype 1 | $p_{SVR(A,1)}$ | N/A | 20-39: 0.54 |
|  |  |  | 40-49: 0.37 |
|  |  |  | 50+: 0.22 |
| Probability of SVR when cirrhosis treated – genotype 1 | $p_{SVR(B,1)}$ | N/A | 20-39: 0.20 |
|  |  |  | 40-49: 0.11 |
|  |  |  | 50+: 0.06 |
| Probability of SVR when treated – non-genotype 1 | $p_{SVR(other)}$ | 0.8 | - |
| Probability of SVR when mild disease treated – non-genotype 1 | $p_{SVR(I,other)}$ | N/A | 20-39: 0.84 |
|  |  |  | 40-49: 0.82 |
|  |  |  | 50+: 0.80 |
| Probability of SVR when moderate disease treated – non-genotype 1 | $p_{SVR(A,other)}$ | N/A | 20-39: 0.73 |
|  |  |  | 40-49: 0.70 |
|  |  |  | 50+: 0.67 |
| Probability of SVR when cirrhosis treated – non-genotype 1 | $p_{SVR(B,other)}$ | N/A | 20-39: 0.43 |
|  |  |  | 40-49: 0.40 |
|  |  |  | 50+: 0.37 |
| Proportion of infections that are genotype 1 [12] | $p_{geno1}$ | 0.5 | 0.45 |
| Proportion mild disease treated per year | $p_{\delta_{1}}$ | 0.02 | 0.02 |
| Proportion moderate disease treated per year | $p_{\delta_{A}}$ | 0.03 | 0.03 |
| Proportion cirrhosis disease treated per year | $p_{\delta_{B}}$ | 0.06 | 0.06 |

**Table S3**. Costs of HCV disease states incurred over the lifetime of the average individual with chronic HCV infection initially aged 20, 35, and 50 years, calculated by each model with model A unit costs used in both models. Where applicable the discount rate used is 3.5%p.a.

| HCV Disease Stage | **Model A** | | | | | | **Model B** | | | | | | **Cost differences**  **(model A – model B)** | | |
| --- | --- | --- | --- | --- | --- | --- | --- | --- | --- | --- | --- | --- | --- | --- | --- |
|  | **Cost (£)** | | | **%** | | | **Cost (£)** | | | **%** | | | **(£)** | | |
|  | 20y | 35y | 50y | 20y | 35y | 50y | 20y | 35y | 50y | 20y | 35y | 50y | 20y | 35y | 50y |
| *Discounted* |  |  |  |  |  |  |  |  |  |  |  |  |  |  |  |
| Mild | 2,520 | 2,450 | 2,270 | 13.1 | 14.3 | 16.4 | 5,290 | 4,420 | 3,430 | 32.8 | 25.6 | 19.7 | -2,770 | -1,970 | -1,160 |
| Moderate | 3,680 | 3,390 | 2,810 | 19.2 | 19.8 | 20.4 | 4,800 | 5,940 | 6,490 | 29.8 | 34.4 | 37.4 | -1120 | -2,550 | -3,680 |
| Cirrhosis | 1,880 | 1,610 | 1,180 | 9.8 | 9.4 | 8.6 | 939 | 1,400 | 1,810 | 5.8 | 8.1 | 10.4 | 941 | 210 | -630 |
| Decompensated cirrhosis | 2,650 | 2,190 | 1,490 | 13.8 | 12.8 | 10.8 | 1,340 | 1,700 | 1,800 | 8.3 | 9.8 | 10.4 | 1,310 | 490 | -310 |
| HCC | 351 | 296 | 209 | 1.8 | 1.7 | 1.5 | 456 | 806 | 1,230 | 2.8 | 4.7 | 7.1 | -105 | -510 | -1021 |
| Transplant and post-transplant | 442 | 357 | 234 | 2.3 | 2.1 | 1.7 | 433 | 670 | 904 | 2.7 | 3.9 | 5.2 | 9 | -313 | -670 |
| Mild (SVR) | 4,050 | 3,800 | 3,370 | 21.1 | 22.2 | 24.4 | 1,700 | 1,210 | 659 | 10.6 | 7.0 | 3.8 | 2,350 | 2,590 | 2,711 |
| Moderate (SVR) | 2,210 | 1,910 | 1,460 | 11.5 | 11.1 | 10.6 | 1,040 | 997 | 875 | 6.5 | 5.8 | 5.1 | 1,170 | 913 | 585 |
| Cirrhosis (SVR) | 1,400 | 1,130 | 763 | 7.3 | 6.6 | 5.5 | 114 | 140 | 142 | 0.7 | 0.8 | 0.8 | 1,286 | 990 | 621 |
| Total | 19,200 | 17,100 | 13,800 |  |  |  | 16,100 | 17,300 | 17,300 |  |  |  | 3,100 | -200 | -3,500 |
| *Undiscounted* |  |  |  |  |  |  |  |  |  |  |  |  |  |  |  |
| Mild | 4,550 | 4,150 | 3,530 | 7.9 | 9.7 | 12.6 | 8,610 | 6,500 | 4,700 | 17.3 | 14.8 | 13.3 | -4,060 | -2,350 | -1,170 |
| Moderate | 9,900 | 8,050 | 5,700 | 17.3 | 18.8 | 20.4 | 14,300 | 14,000 | 11,600 | 28.8 | 31.8 | 32.9 | -4,400 | -5,950 | -5,900 |
| Cirrhosis | 6,690 | 4,800 | 2,840 | 11.7 | 11.2 | 10.2 | 4,350 | 4,650 | 4,450 | 8.7 | 10.6 | 12.6 | 2,340 | 150 | -1,610 |
| Decompensated cirrhosis | 10,500 | 7,180 | 3,890 | 18.4 | 16.8 | 13.9 | 5,790 | 5,530 | 4,590 | 11.6 | 12.6 | 13.0 | 4,710 | 1,650 | -700 |
| HCC | 1,320 | 925 | 525 | 2.3 | 2.2 | 1.9 | 2,900 | 3,280 | 3,360 | 5.8 | 7.5 | 9.5 | -1,580 | -2,355 | -2,835 |
| Transplant and post-transplant | 1,870 | 1,220 | 626 | 3.3 | 2.9 | 2.2 | 2,480 | 2,630 | 2,490 | 5.0 | 6.0 | 7.1 | -610 | -1,410 | -1,864 |
| Mild (SVR) | 9,180 | 7,480 | 5,680 | 16.0 | 17.5 | 20.3 | 5,900 | 3,260 | 1,420 | 11.9 | 7.4 | 4.0 | 3,280 | 4,220 | 4,260 |
| Moderate (SVR) | 7,550 | 5,300 | 3,220 | 13.2 | 12.4 | 11.5 | 4,790 | 3,490 | 2,200 | 9.6 | 8.0 | 6.2 | 2,760 | 1,810 | 1020 |
| Cirrhosis (SVR) | 5,820 | 3,700 | 1,940 | 10.1 | 8.7 | 6.9 | 633 | 561 | 419 | 1.3 | 1.3 | 1.2 | 5,187 | 3,139 | 1,521 |
| Total | 57,400 | 42,800 | 27,900 |  |  |  | 49,800 | 43,900 | 35,200 |  |  |  | 7,600 | -1100 | -7,300 |

**Table S4**. Costs of HCV disease states incurred over the lifetime of the average individual with chronic HCV infection initially aged 20, 35, and 50 years to 3 significant figures, calculated by each model with model B unit costs used in both models. Where applicable the discount rate used is 3.5%p.a.

| HCV Disease Stage | **Model A** | | | | | | **Model B** | | | | | | **Cost differences**  **(model A – model B)** | | |
| --- | --- | --- | --- | --- | --- | --- | --- | --- | --- | --- | --- | --- | --- | --- | --- |
|  | **Cost (£)** | | | **%** | | | **Cost (£)** | | | **%** | | | **(£)** | | |
|  | 20y | 35y | 50y | 20y | 35y | 50y | 20y | 35y | 50y | 20y | 35y | 50y | 20y | 35y | 50y |
| *Discounted* |  |  |  |  |  |  |  |  |  |  |  |  |  |  |  |
| Mild | 2,480 | 2,410 | 2,230 | 15.4 | 16.7 | 19.1 | 4,280 | 3,580 | 2,780 | 30.9 | 23.8 | 18.1 | -1,800 | -1,170 | -550 |
| Moderate | 3,670 | 3,380 | 2,810 | 22.8 | 23.4 | 24.0 | 4,430 | 5,490 | 5,980 | 32.0 | 36.4 | 39.1 | -760 | -2,110 | -3,170 |
| Cirrhosis | 1,850 | 1,590 | 1,160 | 11.5 | 11.0 | 10.0 | 864 | 1,290 | 1,660 | 6.2 | 8.5 | 10.8 | 986 | 300 | -500 |
| Decompensated cirrhosis | 2,620 | 2,170 | 1,480 | 16.3 | 15.0 | 12.7 | 1,330 | 1,680 | 1,790 | 9.5 | 11.2 | 11.7 | 1,290 | 490 | -310 |
| HCC | 343 | 290 | 205 | 2.1 | 2.0 | 1.7 | 447 | 789 | 1,200 | 3.2 | 5.2 | 7.9 | -104 | -499 | -995 |
| Transplant and post-transplant | 437 | 353 | 232 | 2.7 | 2.4 | 2.0 | 428 | 662 | 894 | 3.0 | 4.4 | 5.8 | 9 | -309 | -662 |
| Mild (SVR) | 3,070 | 2,850 | 2,470 | 19.1 | 19.7 | 21.2 | 1,680 | 1,190 | 649 | 12.1 | 7.9 | 4.2 | 1,390 | 1,660 | 1,821 |
| Moderate (SVR) | 1,080 | 954 | 755 | 6.7 | 6.6 | 6.5 | 370 | 355 | 311 | 2.7 | 2.4 | 2.0 | 710 | 599 | 444 |
| Cirrhosis (SVR) | 523 | 444 | 324 | 3.3 | 3.1 | 2.8 | 25 | 31 | 32 | 0.2 | 0.2 | 0.2 | 498 | 413 | 292 |
| Total | 16,100 | 14,400 | 11,700 |  |  |  | 13,900 | 15,100 | 15,300 |  |  |  | 2,200 | -700 | -3,600 |
| *Undiscounted* |  |  |  |  |  |  |  |  |  |  |  |  |  |  |  |
| Mild | 4,480 | 4,090 | 3,470 | 9.5 | 11.4 | 14.7 | 7,000 | 5,280 | 3,810 | 16.2 | 13.7 | 12.2 | -2,520 | -1,190 | -340 |
| Moderate | 9,880 | 8,030 | 5,690 | 20.9 | 22.4 | 24.0 | 13,400 | 12,900 | 10,700 | 31.0 | 33.7 | 34.3 | -3,520 | -4,870 | -5,010 |
| Cirrhosis | 6,600 | 4,740 | 2,800 | 13.9 | 13.2 | 11.8 | 4,040 | 4,300 | 4,090 | 9.4 | 11.2 | 13.1 | 2,560 | 440 | -1,290 |
| Decompensated cirrhosis | 10,400 | 7,110 | 3,850 | 22.0 | 19.9 | 16.3 | 5,740 | 5,480 | 4,550 | 13.3 | 14.3 | 14.6 | 4,660 | 1,630 | -700 |
| HCC | 1,290 | 906 | 514 | 2.7 | 2.5 | 2.2 | 2,840 | 3,220 | 3,290 | 6.6 | 8.4 | 10.5 | -1,550 | -2,314 | -2,776 |
| Transplant and post-transplant | 1,840 | 1,200 | 619 | 3.9 | 3.4 | 2.6 | 2,450 | 2,600 | 2,460 | 5.7 | 6.8 | 7.9 | -610 | -1,400 | -1,841 |
| Mild (SVR) | 7,500 | 5,920 | 4,330 | 15.8 | 16.5 | 18.3 | 5,810 | 3,210 | 1,400 | 13.5 | 8.4 | 4.5 | 1,690 | 2,710 | 2,930 |
| Moderate (SVR) | 3,380 | 2,480 | 1,600 | 7.1 | 6.9 | 6.8 | 1,700 | 1,240 | 781 | 4.0 | 3.2 | 2.5 | 1,680 | 1,240 | 819 |
| Cirrhosis (SVR) | 1,920 | 1,330 | 775 | 4.1 | 3.7 | 3.3 | 142 | 126 | 94 | 0.3 | 0.3 | 0.3 | 1,778 | 1,204 | 681 |
| Total | 47,300 | 35,800 | 23,700 |  |  |  | 43,100 | 38,400 | 31,200 |  |  |  | 4,200 | -2,600 | -7,500 |

**Figure S1**. Discounted QALY losses in cohorts of 1,000 individuals with chronic HCV infection. Top, middle, bottom row: initial age 20, 35, 50 years, respectively. Columns, left to right: model A, model B, difference (model A – model B). Discount rate: 3.5%p.a.


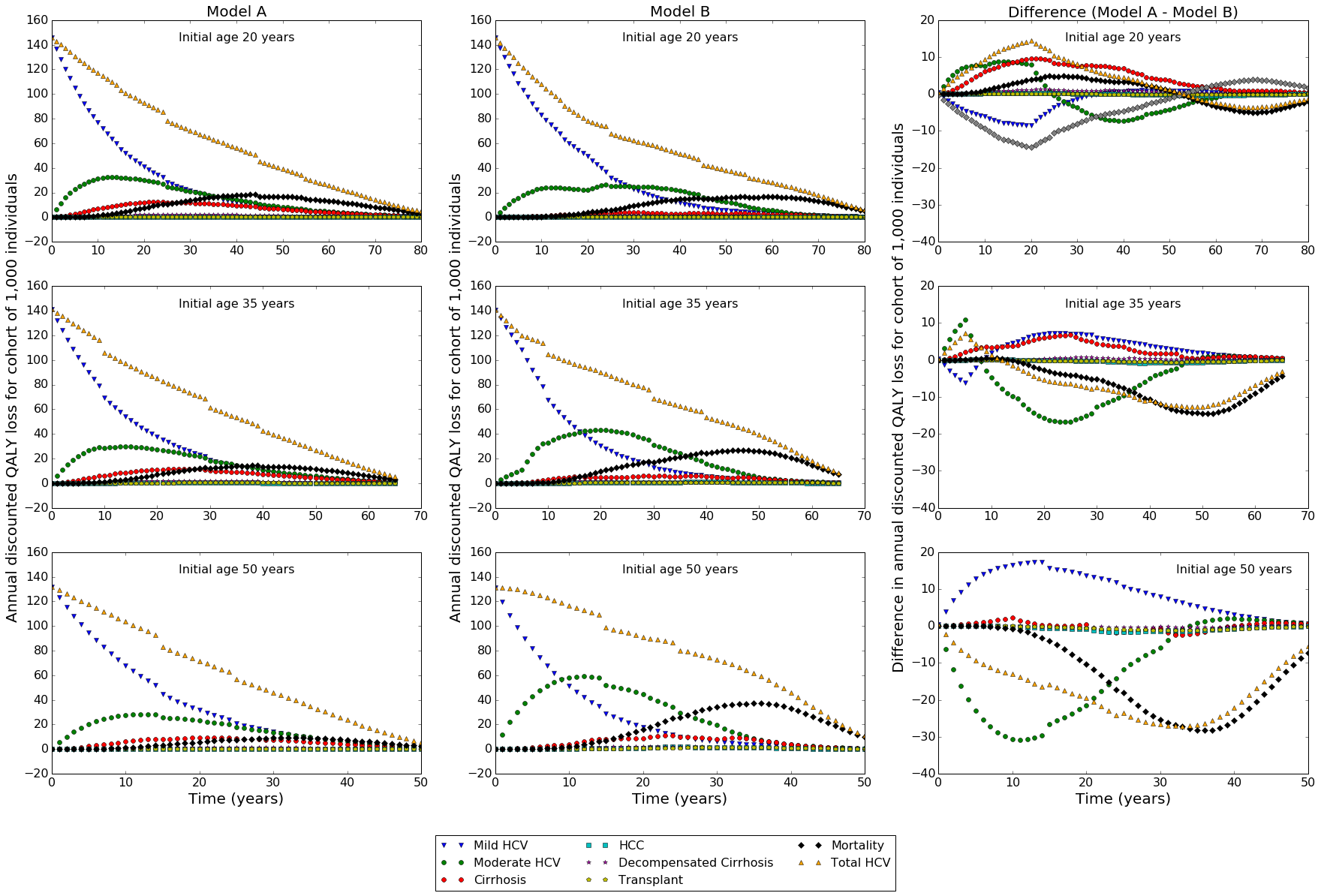


**Figure S2**. Undiscounted QALY losses in cohorts of 1,000 individuals with chronic HCV infection. Top, middle, bottom row: initial age 20, 35, 50 years, respectively. Columns, left to right: model A, model B, difference (model A – model B).


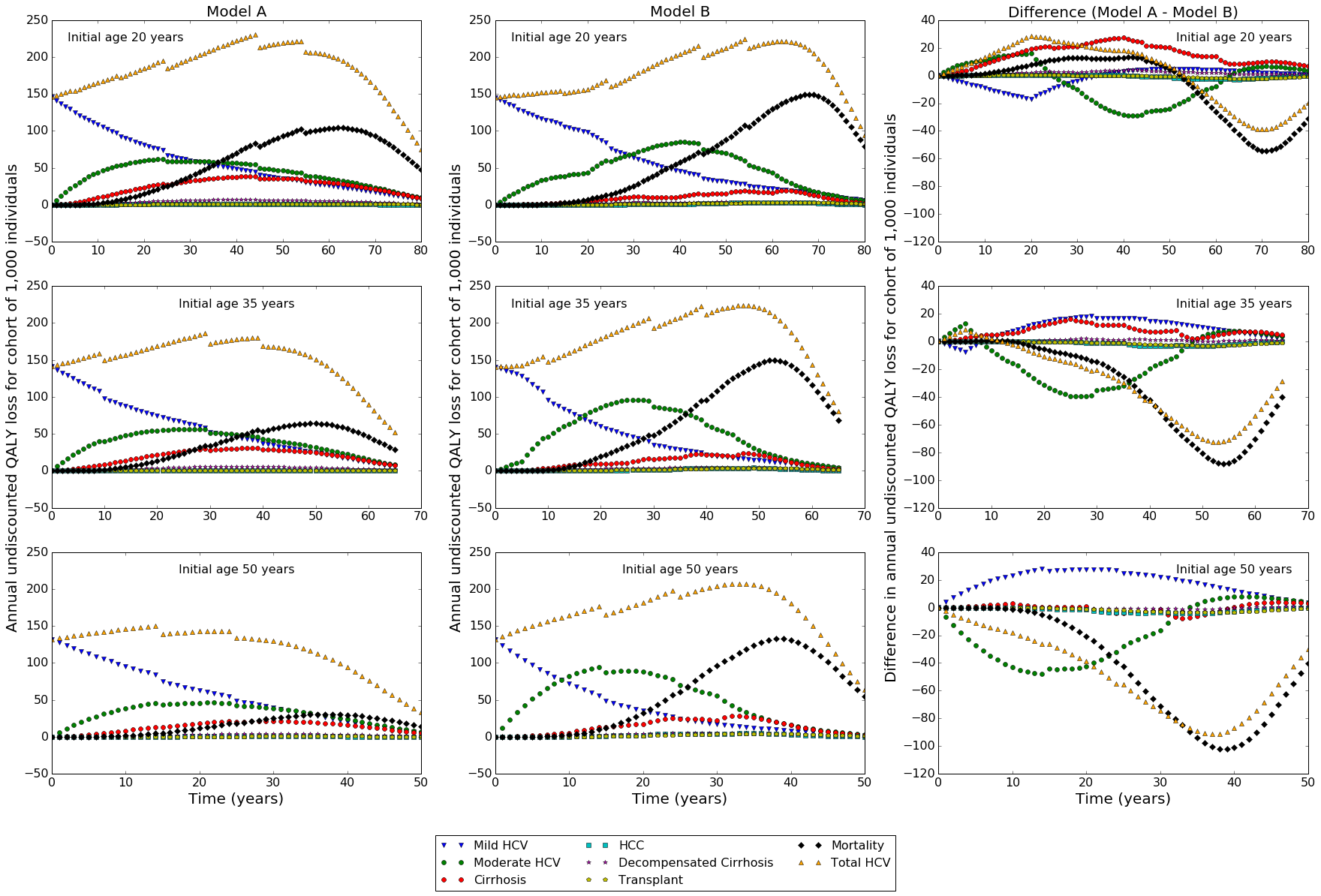


**Figure S3**. Cumulative discounted QALY losses in individuals with chronic HCV infection. Top, middle, bottom row: initial age 20, 35, 50 years, respectively. Columns, left to right: model A, model B, difference (model A – model B). Discount rate: 3.5%p.a.


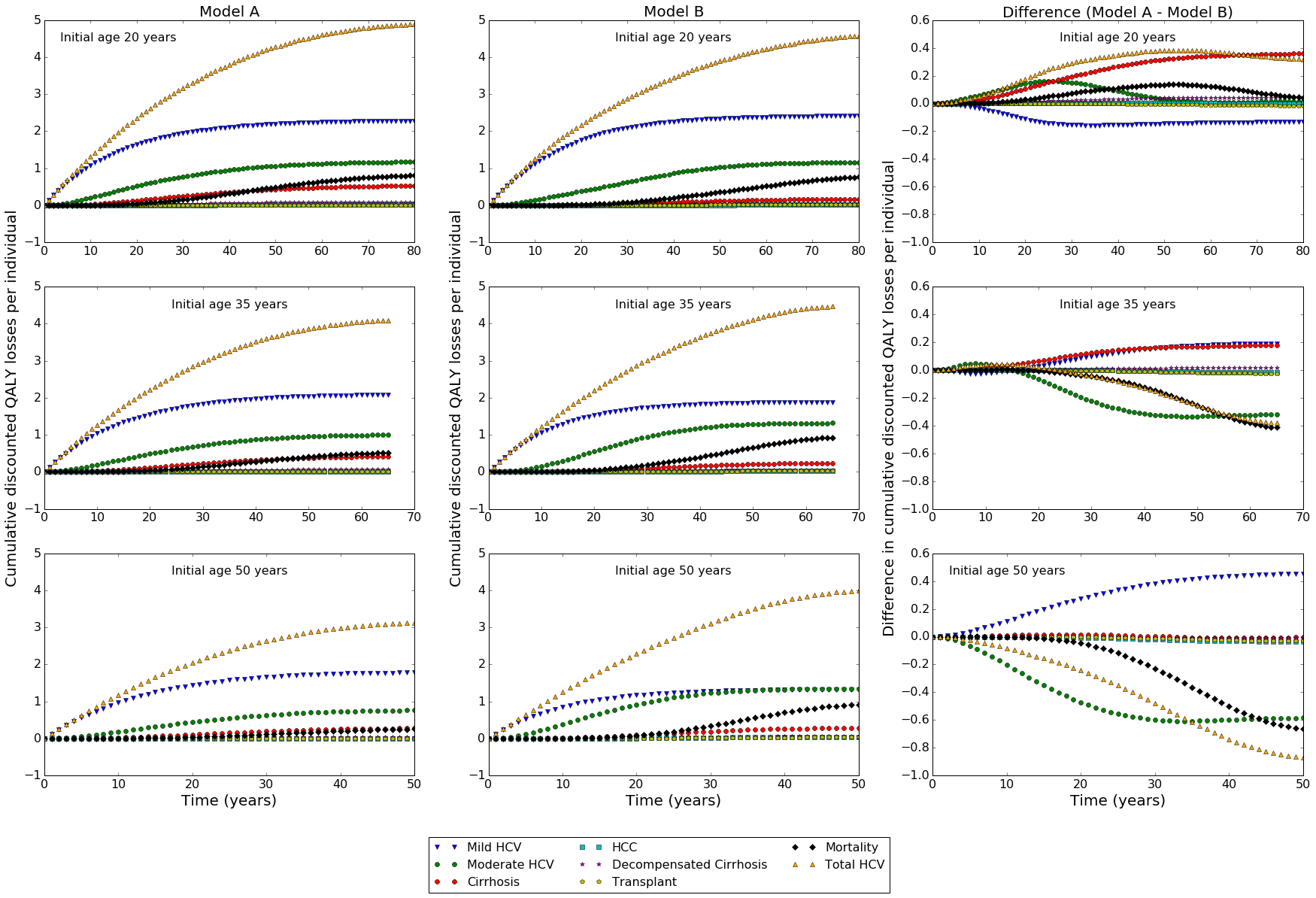


**Figure S4**. Cumulative undiscounted QALY losses in individuals with chronic HCV infection. Top, middle, bottom row: initial age 20, 35, 50 years, respectively. Columns, left to right: model A, model B, difference (model A – model B).


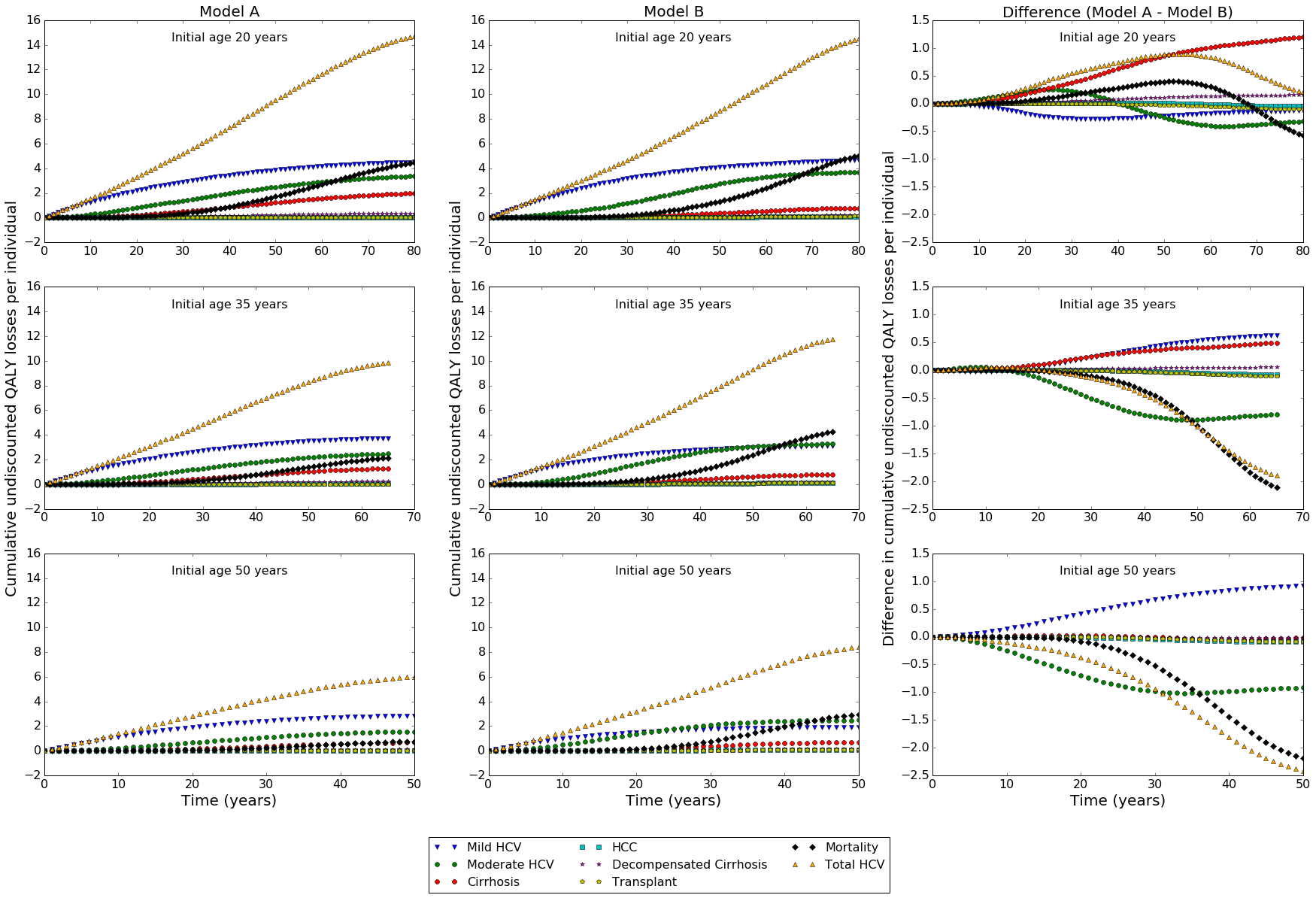


**Figure S5**. Average discounted costs per individual with chronic HCV infection. Top, middle, bottom row: initial age 20, 35, 50 years, respectively. Columns, left to right: model A, model B, difference (model A – model B). Discount rate: 3.5%p.a.


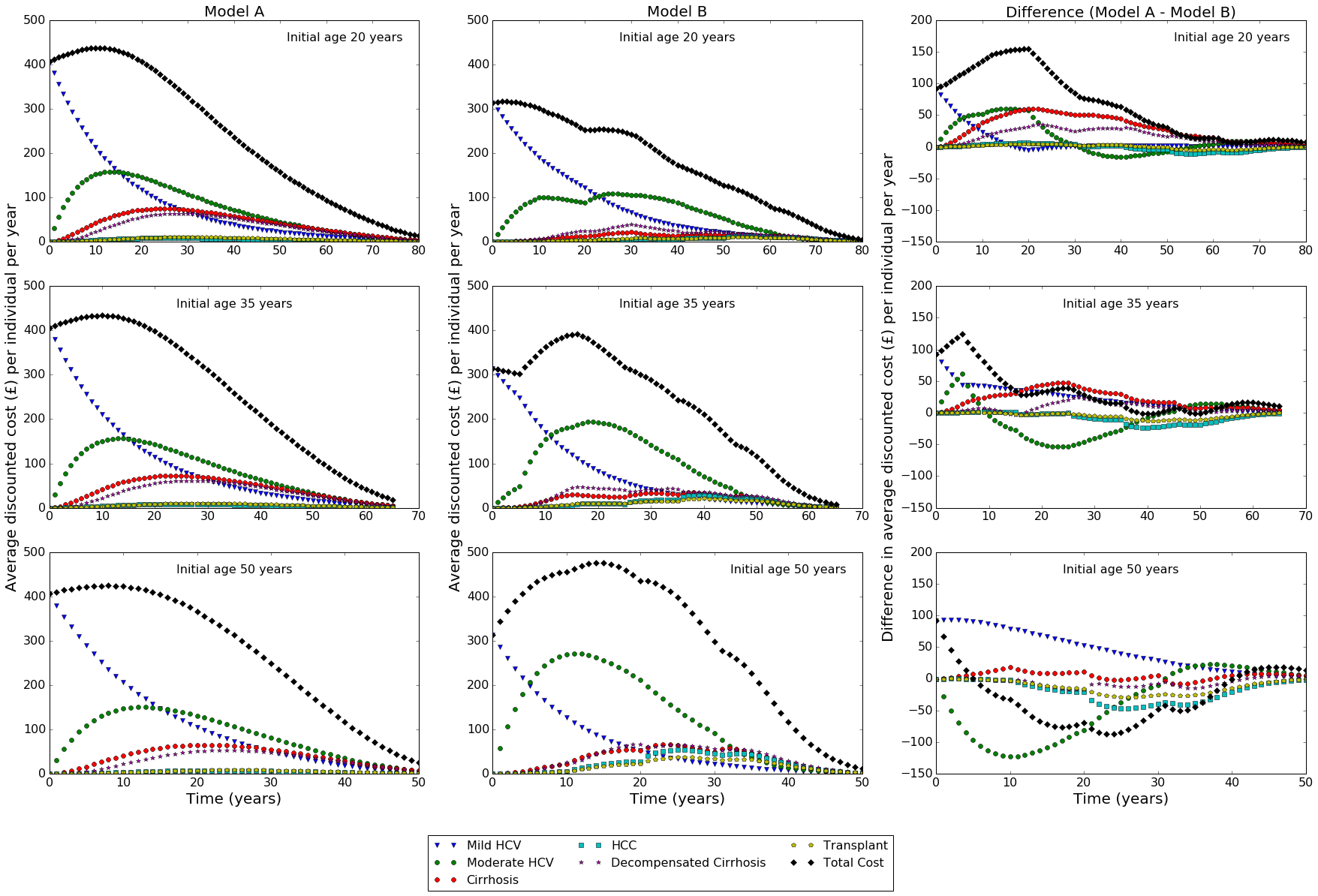


**Figure S6**. Average undiscounted costs per individual with chronic HCV infection. Top, middle, bottom row: initial age 20, 35, 50 years, respectively. Columns, left to right: model A, model B, difference (model A – model B).


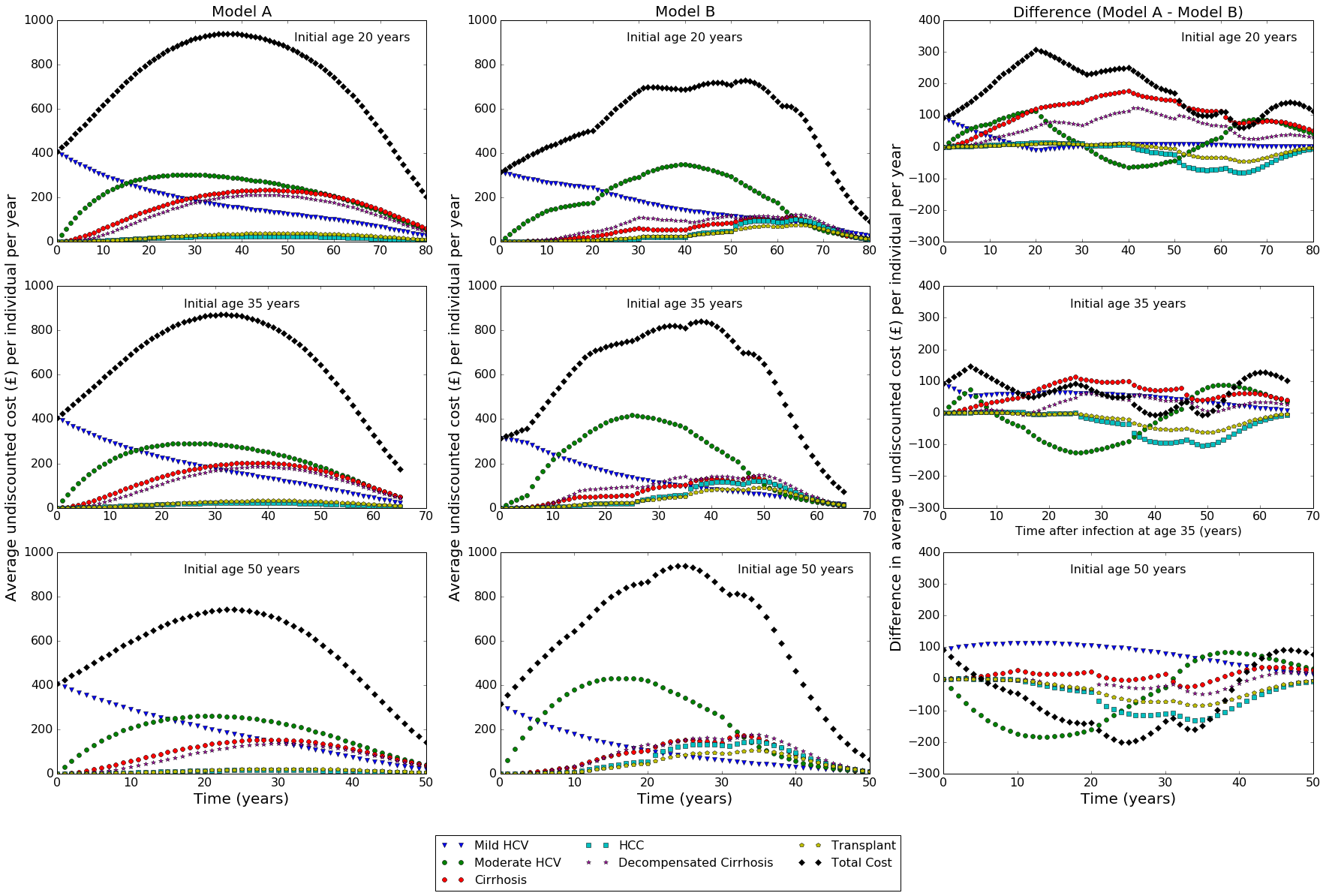


**Figure S7**. Average cumulative discounted costs per individual with chronic HCV infection. Top, middle, bottom row: initial age 20, 35, 50 years, respectively. Columns, left to right: model A, model B, difference (model A – model B). Discount rate: 3.5%p.a.


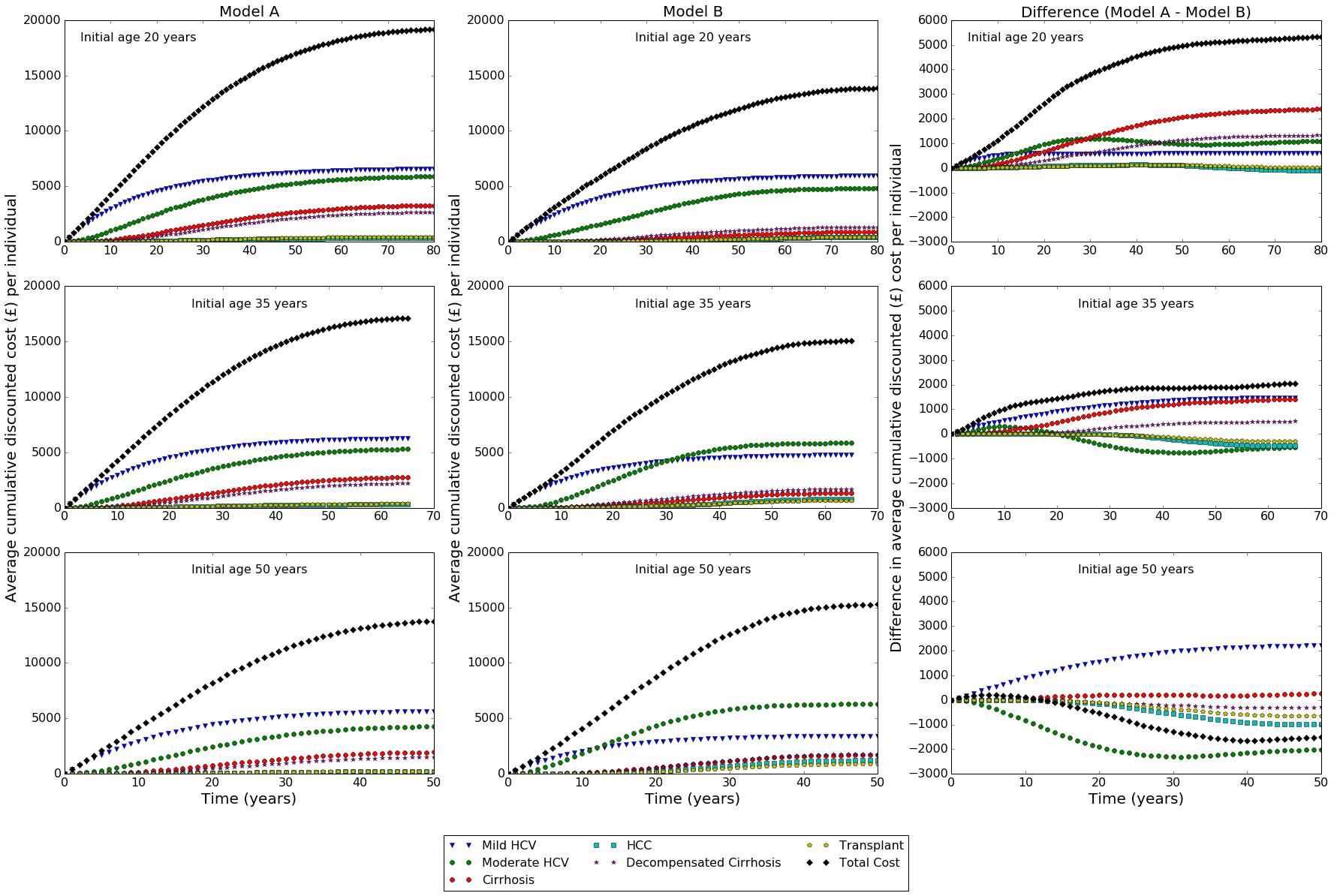


**Figure S8**. Average cumulative undiscounted costs per individual with chronic HCV infection. Top, middle, bottom row: initial age 20, 35, 50 years, respectively. Columns, left to right: model A, model B, difference (model A – model B).


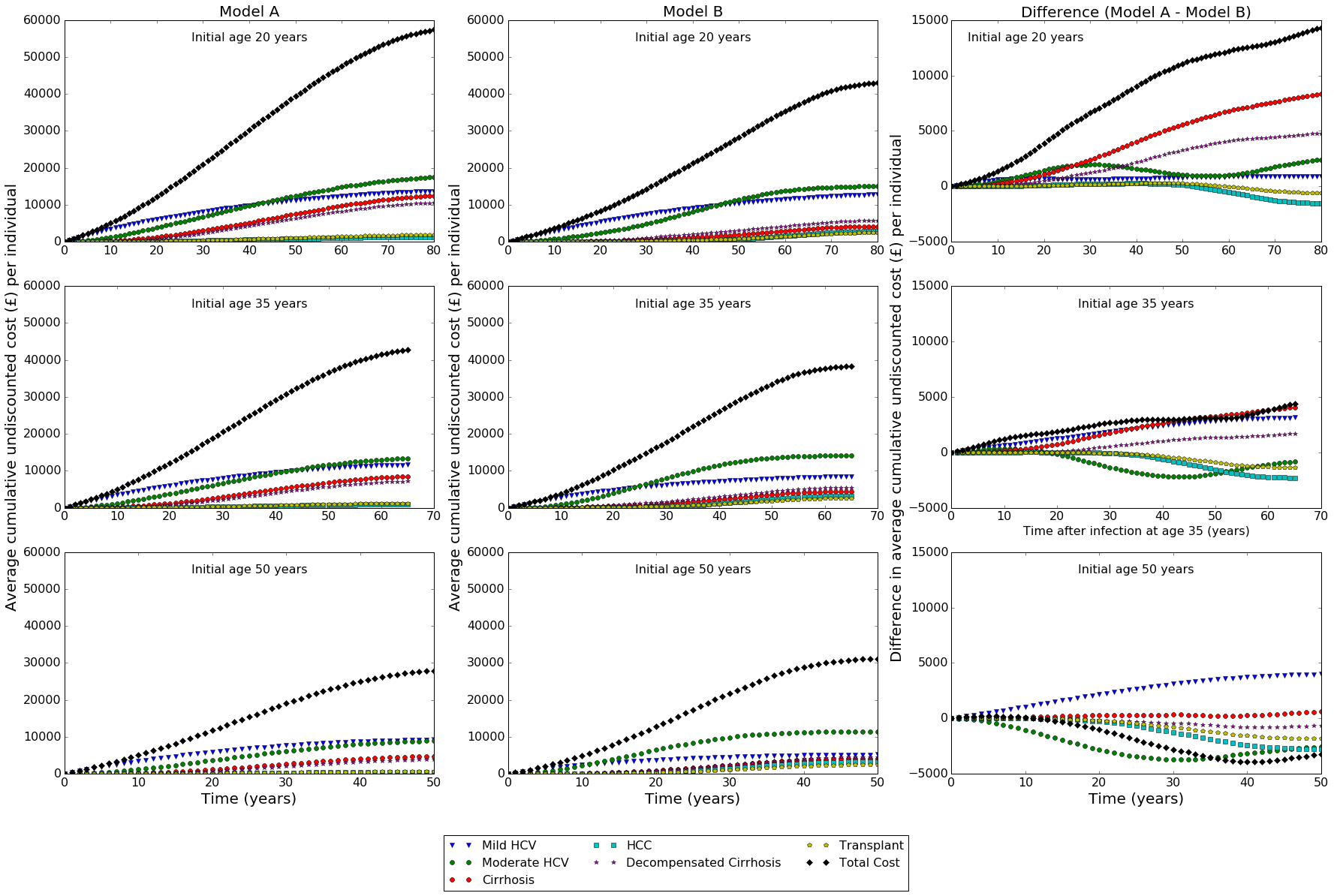

Supplement: Supplementary file 1 [file JVH-25-514-s001.doc]
